# From surgery to functional capacity: muscle strength modifications in women post sleeve gastrectomy

**DOI:** 10.1186/s13102-024-00910-9

**Published:** 2024-05-27

**Authors:** Valentina Bullo, Davide Pavan, Stefano Gobbo, Alessandro Bortoletto, Lucia Cugusi, Andrea Di Blasio, Roberto Pippi, David Cruz-Diaz, Danilo Sales Bocalini, Andrea Gasperetti, Roberto Vettor, Andrea Ermolao, Marco Bergamin

**Affiliations:** 1 Department of Medicine, University of Padova, Via Giustiniani, 2, Padova, 35128 Italy; 2 GymHub S.r.l, Spin-off of the University of Padova, Via O. Galante 67/a, Padova, 35129 Italy; 3 Department of Biomedical Sciences, University of Sassari, Viale San Pietro 43/B, Sassari, 07100 Italy; 4 Department of Medicine and Sciences of Aging, G. d’Annunzio University of Chieti-Pescara, Via dei Vestini 31, Chieti, 66100 Italy; 5 Department of Medicine and Surgery, Healthy Lifestyle Institute, C.U.R.I.A.Mo. (Centro Universitario Ricerca Interdipartimentale Attività Motoria), University of Perugia, Via G. Bambagioni, 19, Perugia, 06126 Italy; 6 Department of Health Sciences, Faculty of Health Sciences, University of Jaén, Jean, Spain; 7 Laboratorio de Fisiologia e Bioquimica Experimental, Centro de Educacao Fisica e Deportos, Universida-Federal do Espirito Santo (UFES), Av. Fernando Ferrari, 514, Goiabeiras, Vitória, Espírito Santo 29075-910 Brazil; 8 Sports and Exercise Medicine Division, Department of Medicine, University of Padova, Via Giustiniani 2, Padova, 35128 Italy; 9 Department of Medicine, Azienda Ospedaliera Padova, University of Padova, Clinica Medica 3, Padova, 35122 Italy

**Keywords:** Muscular strength, Obesity, Sleeve gastrectomy, Weight loss

## Abstract

**Background:**

Severe obesity is characterized by excessive accumulation of fat generating a general health decline. Multidisciplinary treatment of obesity leads to significant weight loss in a few patients; therefore, many incur bariatric surgery. The main purpose of the study is to evaluate changes in functional capacity of people with obesity undergoing bariatric surgery and, in parallel, to correlate pre-surgery functional capacity with weight loss to improve exercise prescription during pre-operatory stage.

**Methods:**

sixty women with diagnosed obesity were included. Maximal oxygen consumption, upper and lower limb strength and level of physical activity were recorded 1 month before and 6 months after sleeve gastrectomy.

**Results:**

significant reduction on body weight (-30.1 kg) and Body Mass Index (-11.4 kg/m^2^) were highlighted after surgery. Absolute grip strength decreased significantly (-1.1 kg), while body weight normalized grip and lower limb strength increased significantly. The level of physical activity increased especially in leisure time (+ 593 METs/week) and active transport (+ 189.3 METs/week). Pre-surgery BMI and age predicted the amount of weight loss after surgery.

**Conclusions:**

Sleeve gastrectomy induces a reduction of muscle strength despite the increase of time spent in physical activity. Further research is necessary to integrate these results with data on body composition, and objective evaluation of physical activity level to define useful information for exercise prescription in terms of surgery pre-habilitation.

**Trial registration:**

Padova University Hospital Board (protocol n. 2027 dated January 12, 2017).

## Introduction

Obesity is classified by the World Health Organization by Body Mass Index (BMI), usually associated with other body parameters such as waist circumference, waist-hip ratio, and waist–height ratio used as an indicator of abdominal obesity and cardiovascular risk [1, 2]. Severe obesity is characterized by excessive accumulation of fat [3] generating an inflammatory state correlated with general health worsening, quality of life and higher risk to develop hypertension, dyslipidemia, diabetes, metabolic syndrome [4], and musculoskeletal impairment affecting mobility and gait capacity [5–7].

Multidisciplinary treatment of obesity leads to significant weight loss in a few patients; hence many incur bariatric surgery [8]. However, adherence to post-operative follow-up is crucial to achieve a long-term effect [9]. Indeed, weight-loss surgery is the most effective procedure for the management of obesity, with greater body weight loss and remission of several comorbidities such as type 2 diabetes mellitus, hypertension, dyslipidemia [10] and consistent reduction in drugs use [11]. Moreover, surgery is a cost-effective treatment for Class II obesity and patients who have no achieved significant weight loss with multidisciplinary treatment [12]. In parallel, the loss of body weight entails changes in physical function. Indeed, after bariatric surgery, a loss of fat-free mass (FFM) accounts for about 31% of weight loss [13], also resulting in strength modification [14]. Several studies in the literature have investigated the variation of muscle strength in small groups of surgically treated people with obesity [15, 16]. However, the aspect of muscle strength has been treated as secondary variable in small samples, with discordant results, using different assessment methods [15], and has been considered sometimes in relative and sometimes in absolute terms. Indeed, it is important to emphasize the difference between muscle strength expressed in relative terms (as body weight normalized) and muscle strength expressed in absolute terms (referring to the maximum tension level that a muscle group can produce) [14, 17]. The first one is an excellent indicator of performance, from a theoretical point of view: in fact, this parameter is close to the concept of muscular power, for example in athletes. On the other hand, the second one is an independent predictor of all-cause mortality and cardiovascular diseases [18]. Moreover, in the case of people with obesity or unfit individuals, absolute strength assumes a high prodromal value as being a predictor of daily living activities performance [19, 20].

In this sense, an interpretative paradox can be incurred when there is an improvement in relative strength, but the reduction in absolute strength could impair some daily living activities. Therefore, even though in people with obesity treated with bariatric surgery the level of physical activity tends to increase, any loss of FFM due to rapid weight loss seems to predispose to long-term weight regain [21], thus following bariatric surgery these patients are considered at high risk for sarcopenic obesity development. Moreover, the impact of sarcopenia in patients after weight loss surgery is still uncertain [22]. Consequently, the assessment of functional capacity before and after surgery is fundamental to check bariatric surgery-induced modifications and draw up the appropriate exercise prescription to facilitate weight loss, while counteracting FFM loss and subsequent weight regain.

The main purpose of the study is to evaluate changes in functional capacity of people with obesity undergoing bariatric surgery while aiming to highlight a correlation between pre-surgery functional capacity and weight loss, which could lead to improvement in exercise prescription during the pre-operatory stage.

## Methods

Women with diagnosed obesity, candidates for bariatric surgery, were recruited from the Sport and Exercise Medicine Division of the University of Padova. Inclusion criteria were: (a) age 18–60 years; (b) BMI (kg/m^2^) > 35; (c) will undergo sleeve gastrectomy (SG) surgery within 1 month from the evaluation; (d) no previous bariatric surgery; (e) able to speak and understand the Italian language; (f) Mini-Mental State Examination higher than 26. Individuals suffering chronic conditions that could affect results (e.g. multiple sclerosis, history of cancer, fibromyalgia), those who would undergo other techniques of bariatric surgery (different from SG), and those who were not able to perform all functional tests were excluded. Written informed consent was obtained prior to enrolment. The study complied with the ethical principles for medical research involving human subjects, as set out in the Helsinki World Medical Association Declaration. The investigation was approved by the Padova University Hospital Board (protocol n. 2027 dated January 12, 2017).

**Fig. 1:**
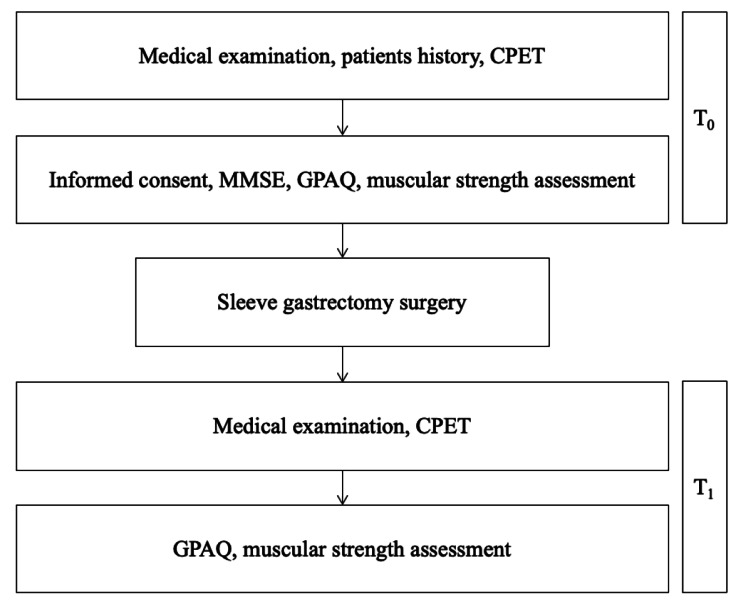
Flow chart of study design. Abbreviation: CPET: cardiopulmonary exercise test; MMSE: mini mental state examination; GPAQ: global physical activity questionnaire; T0: 1-month before surgery; T1: 6-months after surgery

Study design is presented on Fig. 1. Medical history, medical examination, and functional capacity were assessed 1 month before and 6 months after surgery. The Mini-Mental State Examination was administered to all participants at the first visit (before surgery) [23, 24]. Participants’ height and weight were measured with a stadiometer (Ayrton Corporation, Model S100, Prior Lake, MN, USA) and an electronic scale (Home Health Care Digital Scale, Model MC-660, C-7300 v1.1, MO, USA). Height and weight were used to calculate body mass index (BMI). Moreover, the percentage of Excess BMI Loss (%Ex-BMI-L) was computed to determine the success of surgery. Exercise capacity was assessed by incremental cardiopulmonary exercise test (CPET) with gas analysis (Mastercreen CPX Jaeger, carefusion, Hoechberg, Germany) following the modified-Bruce protocol on a treadmill (COSMOS, T170 DE-med model) performed until exhaustion and monitored with Borg Rating of Perceived Exertion scale > 18/20.

Dominant and non-dominant isometric hand grip (HG) strength was evaluated with a calibrated dynamometer (Baseline, Elmsford, NY, USA). The test was performed in a sitting position. The grip handle was adapted to the hand size for a comfortable grab, the elbow was flexed at 90° and adherent to the body, to guarantee the strongest grip measures [25]. Three trials per hand were collected, with 60 s of recovery between trials.

Lower limb muscular strength was evaluated with the multi-joint evaluation system Prima Plus (Easytech, FI, Italy). Patients were seated with the backrest angled at 90°. Belts were placed around the thighs to isolate the movement of knee and ankle joints during the evaluation. The protocol included the evaluation of isometric bilateral knee extension muscular strength, and isokinetic bilateral knee extension and flexion muscular strength. In both tests, the lever fulcrum was aligned with the rotation axis of the knee, and the shin pad was positioned 2 cm above the medial malleolus. The 0° corresponded to the patient-specific maximal knee extension, and the lever was set at 75° of flexion. During the isometric evaluation, the patient had to forcefully push against the pad, maintaining the maximal isometric contraction for 5 s. The trial was performed three times, letting 60 s of rest among them. In the isokinetic test, leg weight was measured and given as input to the software built-in gravity adjustment. The patient performed a maximal knee extension and flexion five times consecutively, without pause between the two movements and the velocity set at 90°/sec. The test was performed three times per each leg, letting 60 s of rest among them. The protocol used for lower limb strength assessment was previously tested and showed high reliability [26, 27]. Outcomes from participants unable to perform the three trials per each type of strength evaluation were excluded from the muscular strength parameters analysis.

Physical activity level was evaluated with Global Physical Activity Questionnaire (GPAQ). The questionnaire domains are about physical activity performed at work, to travel to and from places, and in leisure time. Moreover, it records the daily time spent in sedentary behaviors [28]. For the analysis of “work”, “transport”, “leisure time” and “total” domains, the conversion of weekly minutes of physical activity in METs was performed, following the GPAQ guidelines [28]. Moreover, participants who achieved 600 weekly METs during leisure time were classified as “active”, those who performed less than 600 METs were classified as “not sufficiently active”, while a value of 0 corresponded to the “sedentary” classification.

Statistical analyses were conducted with SPSS (Version 29.0 for Mac, SPSS Inc., Chicago, IL). Data are presented as mean ± standard deviation (SD). The first analysis involved Kolmogorov-Smirnov test to check all the considered variables for normal distribution. Comparison between pre- and post-SG was performed adopting paired T-test variables showing normal distribution; otherwise, the Wilcoxon-signed-rank test was adopted. The effect size (ES) of each outcome measure was computed following the formula ES = (mean pre-value − mean post value)/SD of the mean difference. Interpretation was performed according to Cohen and [29] Sawilowsky’s guidelines [30]. Hierarchical stepwise multiple regression was conducted between pre-surgery physical functional outcomes and weight loss. Normal distribution of residuals was analyzed for predicted variables. Moreover, Pearson’s correlation coefficient (ρ) was calculated between independent variables to determine the level of collinearity. Variables with correlation coefficient higher than 0.8 were excluded from the model. Statistical significance was set at α = 0.05. Sample size calculation was based on the mean values of handgrip detected in a previous study [31]. To this end, the following equation was applied: N = (2(SD2)) × (Zα + Zβ)2)/Δ2.

## Results

Seventy-eight patients were recruited 1 month before SG surgery. Twelve patients were excluded for previous bariatric interventions, and six because underwent to mini-by-pass bariatric surgery. The analysis was applied to 60 women (Table 1).

**Table 1:** Baseline participants’ characteristics before sleeve gastrectomy surgery (mean ± standard deviation)

Characteristic	Mean ± SD	Range [min-max]
Sex (n)	Women (60)	
Age (years)	42.4 ± 11.2	20–60
Height (m)	1.6 ± 0.1	1.5–1.7
Weight (kg)	115.5 ± 15	88–158
BMI (kg/m^2^)	43.9 ± 5.2	36.1–56
MMSE (score)	29.2 ± 1.1	26–30
Days from surgery to post-test	216.5 ± 6.8	204–237
Comorbidities (type)	Pre-diabetes (10), DMT2 (10), hypothyroidism (13), dyslipidemia (16), IPTS (21), asthma (6), OSAS (4), musculoskeletal disorders (17), other (16)	
Comorbidities (num)	No com. (12), 1 com. (16), 2 com. (12), 3 com. (9), 4 com. (7), 5 com. (2), > 6 com. (2)	
Drugs (num)	No drug (18), 1 drug (14), 2 drugs (12), 3 drugs (5), 4 drugs (4), 5 drugs (3), > 6 drugs (4)	
Obesity class (%)	Class II: 15 (25%) Class III: 45 (75%)	

Abbreviation: BMI: body mass index; com: comorbidities; DMT2: type 2 diabetes mellitus; IPTS: hypertension; MMSE: mini mental state examination; num: number; OSAS: obstructive sleep apnea syndrome; Class II: BMI 35-39.9; Class III: BMI > 40;

The success of bariatric surgery is commonly accepted as a % of excess BMI loss (%Ex-BMI-L) higher than 50%. After 6 months, 3% of patients were classified as class III of obesity, 20% as class II and 49% as class I, while 28% of patients were overweight. The mean %Ex-BMI-L was 62.3 ± 0.1%. Moreover, an overall reduction of comorbidities and drugs use was highlighted. As expected, bariatric surgery led to a significant decrease in body weight and BMI (Table 2).

**Table 2:** Changes of anthropometric parameters and health status after sleeve gastrectomy

	Pre (m ± SD)	Post (m ± SD)	Δ (m ± SD) [C.I. 95%]	Δ (%)	ES [C.I. 95%]
Weight (kg)	115.5 ± 15	85.4 ± 11.4	-30.1 §	-26%	
BMI (kg/m^2^)	43.9 ± 5.2	32.4 ± 4.2	-11.4 ± 6.2 [-13; -9.9] **	-26%	-1.9 [-2.3; -1.4]
Comorbidities (type)		Pre-diabetes (2), DMT2 (5), hypothyroidism (13), dyslipidemia (8), IPTS (9), asthma (7), OSAS (2), musculoskeletal disorders (12), other (11)			
Comorbidities (num)		No com. (24), 1 com. (14), 2 com. (10), 3 com. (10), 4 com. (1), 5 com. (1), > 6 com. (0)			
Drugs (num)		No drug (23), 1 drug (18), 2 drugs (8), 3 drugs (7), 4 drugs (3), 5 drugs (0), > 6 drugs (1)			
Obesity class (%)		Overweight: 17 (28%) Class I: 29 (48%) Class II: 12 (20%) Class III: 2 (3%)			

* *p* < 0.05; **p < 0.001; §: Wilcoxon test

Abbreviation: BMI: body mass index; C.I.: confidence interval; ES: effect size Cohen’s *d*; m ± SD: mean ± standard deviation; Δ: absolute change as “post - pre”; com: comorbidities; DMT2: type 2 diabetes mellitus; IPTS: hypertension; OSAS: obstructive sleep apnea syndrome; Overweight: BMI 25-29.9; Class I: BMI 30-34.9; Class II: BMI 35-39.9; Class III: BMI > 40

Upper limb absolute muscular strength decreased significantly after surgery, while relative muscular strength increased significantly. Lower limb absolute muscular strength recorded no statistically significant changes, while relative muscular strength increased significantly. Absolute VO_2_max decreased significantly after SG, while relative VO_2_max increased significantly. After surgery, a significant overall increase in total weekly METs, leisure time METs, and active transport METs was underlined. No significant modifications were found in working activity METs and daily sedentary behaviors (Table 3).

**Table 3:** Changes of muscular strength, maximal oxygen consumption and level of physical activity after sleeve gastrectomy

	Pre (m ± SD)	Post (m ± SD)	Δ (m ± SD) [C.I. 95%]	ES [C.I. 95%]
HG dom (kg)	24.8 ± 5.3	23.7 ± 4.3	-1.1 ± 3.6 [-2;-0.2] *	-0.3 [-0.6;0]
HG no-dom (kg)	22.5 ± 4.9	21.6 ± 4.3	-0.8 ± 3.1 [-1.6;0] *	-0.3 [-0.5;0]
R-HG dom (kg/kg)	0.2 ± 0	0.3 ± 0.1	0.1 ± 0 [0.1;0.1] **	1.5 [1.1;1.9]
R-HG no-dom (kg/kg)	0.2 ± 0	0.3 ± 0.1	0.1 ± 0 [0.1;0.1] **	1.6 [1.2;1.9]
ISOmax (Nm)	221.8 ± 67.5	223.8 ± 59	2.0 ± 50.3 [-11;15]	0.0 [-0.2;0.3]
ISOmed (Nm)	182.0 ± 57.4	190.1 ± 51	8.2 ± 43.8 [-3.1;19.5]	0.2 [-0.1;0.4]
EXT (Nm)	144.0 ± 42.1	145.7 ± 35.2	1.6 ± 36.9 [-7.9;11.2]	0.0 [-0.2;0.3]
FLEX (Nm)	77.9 ± 22.8	75.6 ± 17	-2.3 ± 18 [-6.9;2.4]	-0.1 [-0.4;0.1]
R-ISOmax (Nm)	1.9 ± 0.6	2.6 ± 0.8	0.7 ± 0.5 [0.5;0.8] **	1.3 [0.9;1.6]
R-ISOmed (Nm)	1.5 ± 0.5	2.2 ± 0.7	0.6 ± 0.5 [0.5;0.7] **	1.4 [1;1.7]
R-EXT (Nm)	1.2 ± 0.4	1.7 ± 0.4	0.5 ± 0.4 [0.4;0.6] **	1.2 [0.9;1.5]
R-FLEX (Nm)	0.7 ± 0.2	0.9 ± 0.2	§	
VO_2_max (L/min)	2.2 ± 0.3	1.9 ± 0.3	-0.3 ± 0.3 [-0.4;-0.2] **	-1.1 [-1.4;-0.8]
R-VO_2_max (ml/min/kg)	19.4 ± 2.7	22.4 ± 4.5	2.9 ± 3.8 [2;3.9] **	0.8 [0.5;1.1]
METs work (M/w)	599.7 ± 1070.3	746.0 ± 1428.4		
METs transport (M/w)	282.0 ± 655.9	471.3 ± 737.3	§	
METs leisure time (M/w)	403.0 ± 650	996.0 ± 936.6	593.0 ± 1074 [315.5;870.5] **	0.6 [0.3;0.8]
METs total (M/w)	1284.7 ± 1475.7	2213.3 ± 1938.5	928.7 ± 2245.9 [348.5;1508.8] **	0.4 [0.1;0.7]
SED (min/day)	317.3 ± 183.3	337.3 ± 209.4		

* *p* < 0.05; *p* < 0.001; §: Wilcoxon test

Abbreviation: C.I.: confidence interval; ES: effect size Cohen’s *d*; m ± sd: mean ± standard deviation; Δ: absolute change as “post - pre”; HG: handgrip test; dom: dominant hand; no-dom: non dominant hand; ISOmax: maximal isometric strength of knee extensors; ISOmed: mean isometric strength of knee extensors for 5 s; EXT: maximal isokinetic strength of knee extensors; FLEX: maximal isokinetic strength of knee flexors; R-: relative; VO_2_max: maximal oxygen consumption; METs: weekly metabolic equivalent of task; M/w: weekly METs; min/day: minute per day; SED: sedentary activity

Pearson correlation of pre-surgery outcomes identified seven independent variables: BMI, age, HG strength of dominant hand (HGdom), isometric maximal strength of knee extensors (ISOmax), VO_2_max, weekly total METs and minute of daily sedentary behaviors. Hierarchical stepwise multiple regression was conducted with weight loss as dependent variable. Results indicated BMI and age as predictor variables for weight loss 6 months after surgery (Table 4). In details, BMI alone was able to predict the 34% of variance, while age the 21%. The overall model predicts the 47% of variance. The other variables increase the model of another 7% of variance, with no statistical significance.

**Table 4:** Hierarchical stepwise multiple regression examining the effect of pre-surgery outcomes on weight loss

Variables	β	S.E. β	t	C.I. 95%	ρ	*R*	*R* ^2^	Adjusted *R*^2^	F
BMI	-0.78	0.15	t(57) = -5.28**	-1.07 to -0.48	-0.59**	0.57	0.34	0.33	30.4**
Age	0.25	0.07	t(57) = 3.68**	0.11 to 0.38	0.46**	0.46	0.21	0.2	15.48**
Final model						0.69	0.47	0.45	25.24**

* *p* < 0.05; ** *p* < 0.001; Abbreviation: BMI: Body Mass Index; C.I.: confidence interval; F: Welch’s F Test; R: correlation coefficient; R2: multiple correlation coefficient; S.E.: standard error; t: value of the test statistics from the t distribution; β: individual contribution of predictor to the model; ρ: Pearson correlation with weight loss

## Discussion

The main aim of this study was to evaluate the functional capacity of patients with obesity before and after sleeve gastrectomy. A significant reduction in body weight and BMI was found after surgery. Also, absolute muscular strength of upper limb and maximal oxygen consumption decreased, while all the assessed parameters, corrected by body weight, increased after SG. Those findings are partially in accordance with previous studies [14, 31].

The evaluation of muscular strength, i.e. HG test, is commonly diffused in clinical practice [32]. In fact, low HG strength is associated with sarcopenia, functional impairment, and disabilities [33]. At the same time, cardiorespiratory fitness is relevant for health evaluation and risk of post-surgery complications. Absolute muscle strength of upper limb and absolute VO_2_max decreased significantly after surgery, whereas relative strength and VO_2_max increased. These findings are probably related to a bariatric surgery induced loss of FFM [34]. On the other hand, whilst unaltered absolute muscle strength of the lower limb was found after SG, relative muscle strength significantly increased 6 months after surgery, as previously reported by Handrigan and colleagues [35]. Although absolute strength increased, no statistical significance and small ES were showed.

Cardiorespiratory fitness hinges on the collaborative performance of the cardiovascular, pulmonary, and skeletal muscle systems [36]. The decrease in absolute VO_2_max may stem from various factors, such as FFM reduction [37]. In fact, during the early stages of bariatric surgery weight loss primary involves the loss of FFM [38] that influence changes in functional capacity. Probably, muscle quality and its efficiency are impaired by fat infiltration into muscular tissue [39], but the reduction of fat mass may reduce myosteatosis as well, with muscular strength improvement [22]. Moreover, also muscle structure may influence muscle strength [40]; after surgery, the muscle size of the quadriceps femoris is generally adapted to the total body weight, and the quick weight loss without specific resistance training induces a reduction of muscle cross-sectional area [41]. In our study, despite the increase in postoperative physical activity reported by the patients, we did not know the type of exercise and the real stimuli given to the muscle and therefore the effects on muscle strength. Furthermore, body composition of the patients was not included in data collection, hence an in-depth analysis of muscle strength change, in relation to the real loss of fat mass and muscle mass, was not included.

The main mechanism involved in weight loss is the balance between energy intake and energy expenditure. The role of bariatric surgery is to reduce energy intake, inducing a negative balance to facilitate energy deficit and weight loss. Our study showed a spontaneous increase in METs spent in transport activity and leisure time physical activity after bariatric surgery; indeed, 76.7% of patients reached the recommended minimum level of physical activity (600 METs/week) [42]. On the other hand, time spent in sedentary behaviors do not change after surgery. Probably, most of the time engaged in sedentary activities was work-related. Our results are partially confirmed by previous study. Generally, bariatric surgery patients non increase physical activity and, who improved the level did not reach the recommendations [43].

The second objective of the study was to evaluate whether anthropometrical characteristics, physical fitness, and physical activity level can predict the amount of weight loss following surgery. In fact, Jabbour and colleagues ^39^ highlighted in their review that further studies are required to explore the most effective and suitable form of exercise prescription prior to bariatric surgery while considering physical and psychological limitations of people with obesity. Among the seven parameters examined, only BMI and age significantly predicted a great weight loss 6 months after surgery, with better prediction in young patients with higher BMI. Although pre-surgery physical fitness was no underlined as a predictor of great weight loss, being physically active after surgery dictates better weight loss results [44] and weight regain avoidance [45].

Often, people with obesity tend to be inactive due to a lack of benefit about short-term detectable weight loss. Specific counseling programs should be promoted before and after bariatric surgery to enable a lifestyle change, and therefore a long-term surgery efficacy. In fact, preoperative attitudes to physical activity (people perceiving more exercise benefits, having more confidence with exercise) and behaviors (increasing exercise before surgery) predict higher postoperative physical activity [46].

This study has several limitations. Firstly, body composition analysis was not performed due to a h of dual-energy x-ray absorptiometry weight support. Results in terms of changes in muscle strength, and the effect of FFM on weight loss, cannot be explored in depth without available data. Secondly, physical activity was not recorded using objective measures, thus results on modifying physical activity behaviors appear unclear. The type of exercise performed by the participants could be explored to determine if different stimuli influence differently the changes in physical fitness and weight loss. Finally, there is no detailed dietary record, to pinpoint the effect of the negative energy balance on the overall and partitioned weight loss.

## Conclusion

Sleeve gastrectomy reduces body weight and BMI, with better results in young patients with great BMI. Evaluation of functional capacity showed a decrease of absolute cardiopulmonary capacity and muscular strength of upper limb, whereas those very two variables increased when corrected accounting for body weight. Furthermore, the level of physical activity increased especially in leisure time and active transport, suggesting the beginning of a lifestyle change. Further research is necessary to integrate these results with data on body composition, and objective evaluation of physical activity level to define useful information for exercise prescription in terms of surgery pre-habilitation.

## Data Availability

Materials described in the manuscript, including all relevant raw data, will be freely available to any scientist. For sharing data contact corresponding author stefano.gobbo@unipd.it.
